# Novel *SCN5A* Frameshift Mutation in Brugada Syndrome Associated With Complex Arrhythmic Phenotype

**DOI:** 10.3389/fgene.2019.00547

**Published:** 2019-06-06

**Authors:** Emanuele Micaglio, Michelle M. Monasky, Giuseppe Ciconte, Gabriele Vicedomini, Manuel Conti, Valerio Mecarocci, Luigi Giannelli, Federica Giordano, Alberto Pollina, Massimo Saviano, Paolo R. Pozzi, Chiara Di Resta, Sara Benedetti, Maurizio Ferrari, Vincenzo Santinelli, Carlo Pappone

**Affiliations:** ^1^Arrhythmology Department, IRCCS Policlinico San Donato, Milan, Italy; ^2^Genomic Unit for the Diagnosis of Human Pathologies, Division of Genetics and Cellular Biology, IRCCS San Raffaele Hospital, Milan, Italy; ^3^Vita-Salute San Raffaele University, Milan, Italy; ^4^Laboratory of Clinical Molecular Biology and Cytogenetics, IRCCS San Raffaele Hospital, Milan, Italy

**Keywords:** Brugada syndrome, sudden cardiac death, genetic testing, mutation, *SCN5A*, sodium channel, arrhythmia, atrial fibrillation

## Abstract

In this case report, we characterize a novel inherited frameshift mutation c.4700_4701del (p.Phe1567Cysfs^*^221) in a single copy of the *SCN5A* gene and its association with Brugada syndrome (BrS). The proband experienced a life-threatening ventricular arrhythmia successfully treated with DC-shock and he also suffered from supraventricular tachycardia. Ajmaline test confirmed the BrS diagnosis. No other mutation nor low frequency variants in the other 23 analyzed genes were detected. The same mutation was found in the father and sister, who were both diagnosed with BrS. We hypothesize that this mutation could be responsible for BrS and potentially linked to supraventricular tachycardias. Further studies are needed to confirm this observation and to assess the clinical relevance of this mutation, in terms of risk-stratification.

## Introduction

The Brugada syndrome (BrS) is characterized by a coved-type ST-segment elevation in the right precordial leads on the electrocardiogram (ECG) and an increased risk of sudden cardiac death (SCD). Diagnosis requires the presence of a type 1 BrS pattern on the ECG that occurs either spontaneously or after administration of a sodium channel blocking agent, such as ajmaline (Antzelevitch et al., 2016), which reveals the type 1 pattern. The arrhythmias that occur in BrS can be eliminated using transcatheter radiofrequency (RF) ablation of the arrhythmogenic substrate (AS) that is usually found in the epicardial surface of the right ventricle (RV). Ajmaline may be used to fully visualize the extent of the AS to improve the success of the procedure.

Brugada syndrome is inherited as an autosomal dominant condition with incomplete penetrance. The most commonly associated genetic mutations are found in the *SCN5A* gene, thought to be responsible for about 15–30% of BrS cases (Kapplinger et al., 2010). However, no genetic mutations of any kind are detected in the majority of BrS patients, perhaps due to both locus heterogeneity and large genomic rearrangements only detectable with other genetic analyses. The need exists to further understand the genetics of BrS, especially for the high rate of asymptomatic individuals who could undergo pre-symptomatic non-invasive genetic testing and, if mutated, be recommended for a specific arrhythmologic follow-up.

## Case Presentation

Written informed consent of human subjects included in this case report was obtained for their participation in the study and for publication of this case report. The procedures employed were reviewed and approved by the local Ethics Committee. The proband is a 28-year-old male with a history of recurrent syncope episodes. A 12-lead ECG performed at the age of 27 years demonstrated a type 3 BrS pattern. After a couple of weeks, the proband underwent a 12-lead ECG that demonstrated atrioventricular nodal reentrant tachycardia (AVNRT). One month later, he was admitted to the emergency room elsewhere because of a life-threatening ventricular arrhythmia successfully treated with DC-shock. After sinus rhythm restoration, a type 1 BrS pattern was noticed. The patient underwent an electrophysiological study (EPS) and was found to be inducible. Therefore, he received a subcutaneous ICD.

**FIGURE 1:**
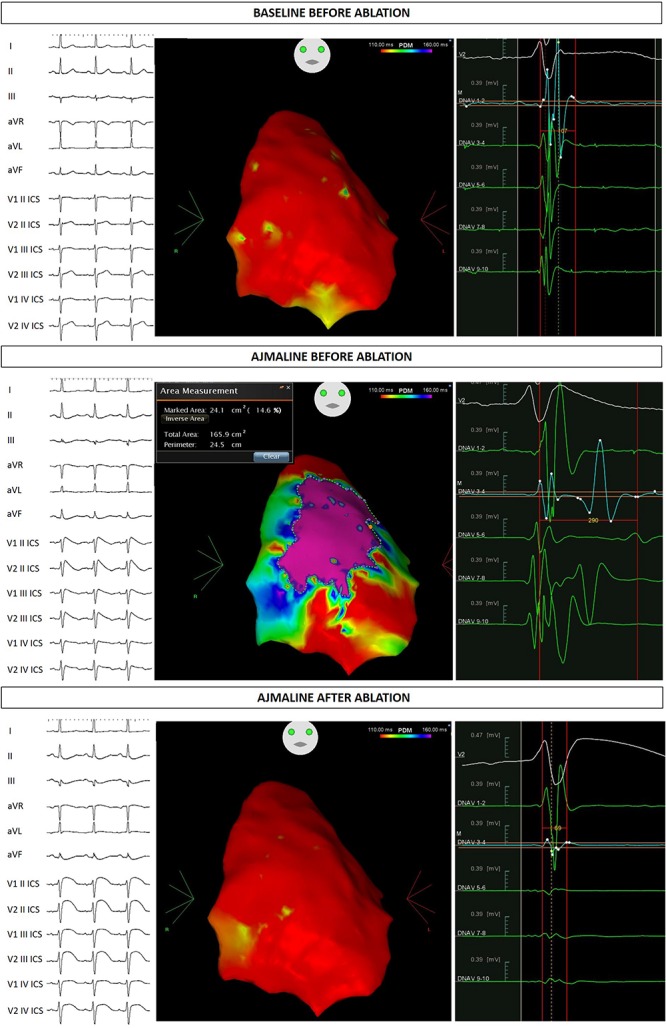
Proband ECG and potential duration map at baseline, after ajmaline administration, and after ablation.

At this stage he came to our attention. All the performed clinical tests (metabolic assessment, EEG, anion-cation concentration, hormonal functions) were negative. Next, an ajmaline test confirmed BrS. Thereafter, he underwent catheter ablation of AVNRT, and the AS responsible for BrS (Figure 1). The EPS and ajmaline test were repeated after ablation and were both negative. A control echocardiogram showed no pericardial effusion.

### Genetic Studies

**FIGURE 2:**
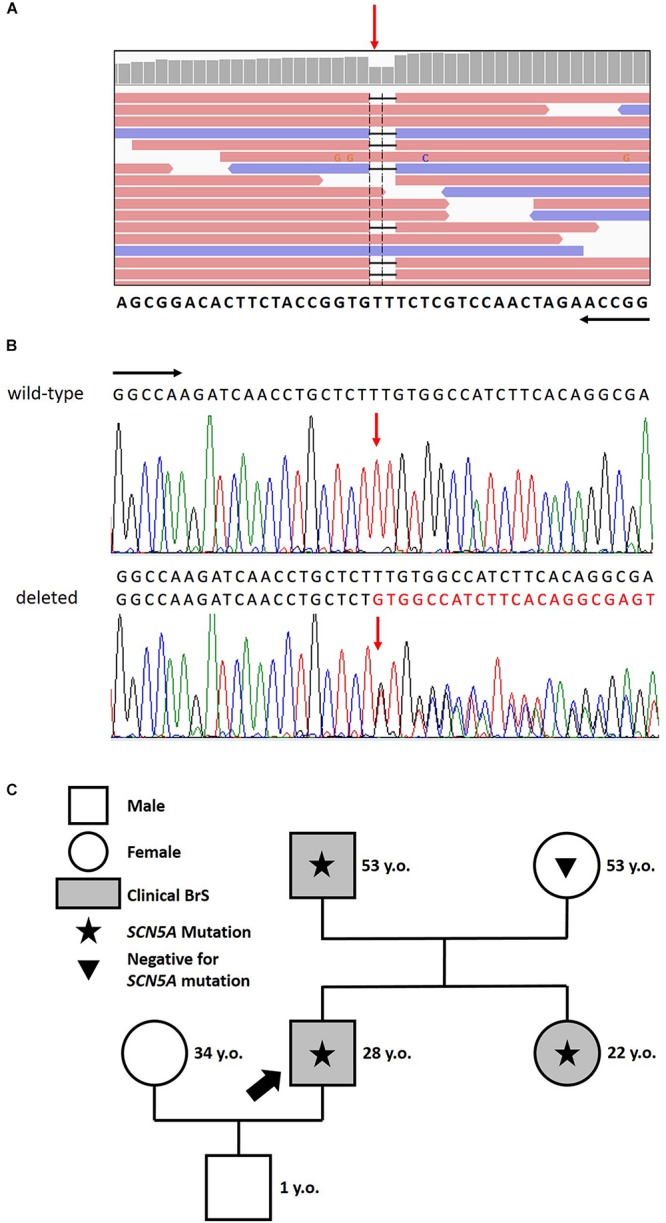
**(A)** Identification of the heterozygous c.4700_4701del deletion by next generation sequencing. NGS paired-end reads loaded in the IGV genome browser. The gene sequence is in the reverse orientation on the chromosome. **(B)** Identification of the heterozygous c.4700_4701del deletion by sanger sequencing. Sanger sequencing confirmation of the deletion compared with the wild-type sequence. The wt (black) and mutated (red) sequences are reported. The position of the two bases deleted is indicated by the red arrow. Black arrows indicate the direction of the gene. **(C)** Family pedigree. Proband identified with an arrow. Square: male; Circle: female; Shaded: clinically affected by Brugada syndrome; Star: molecularly confirmed *SCN5A* mutation: Triangle: genetically tested and negative for *SCN5A* mutation.

After careful genetic counseling, we decided to perform a genetic analysis of genomic DNA extracted from saliva and study 24 genes known to be causative of BrS (*ABCC9, AKAP9, CACNA1C, CACNA2D1, CACNB2, DSG2, GPD1L, HCN4, KCND2, KCND3, KCNE3, KCNE5, KCNH2, KCNJ8, MOG1, PKP2, RANGRF, SCN5A, SCN10A, SCN1B, SCN2B, SCN3B, SEMA3A, TRPM4*). This analysis demonstrated that the proband carries the novel mutation c.4700_4701del (p.Phe1567Cysfs^*^221) in a single copy of the *SCN5A* gene (LOVD^1^). No other mutation nor low frequency variants in the other 23 analyzed genes were detected. The sequence variation can be found in Figure 2.

### Assessment of Family Members

**FIGURE 3:**
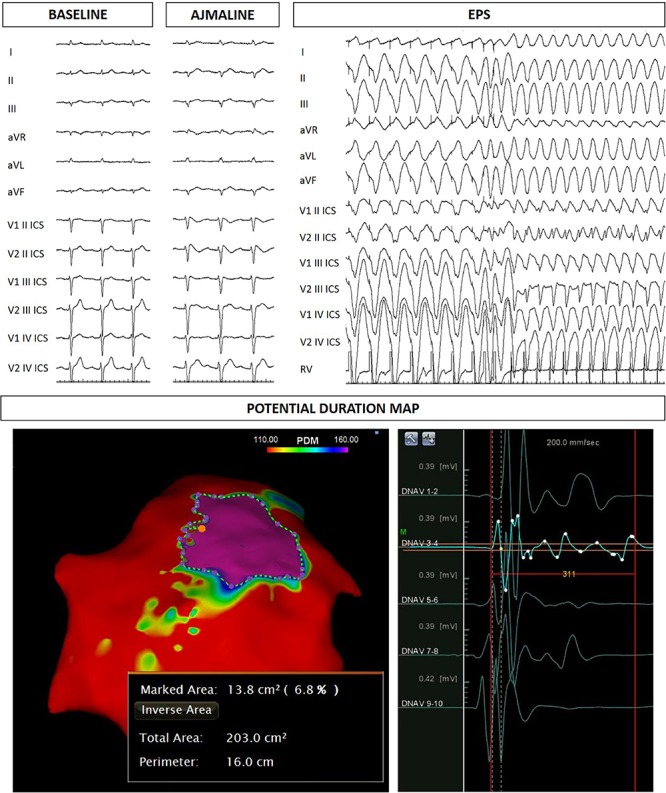
Father of the proband: ECG at baseline, after ajmaline administration, VT/VF inducibility during EPS. Epicardial arrhythmogenic substrate.

The proband’s father is an otherwise healthy 53-year-old man who suffered from atrial fibrillation since he was 23 years old. At the age of about forty, he was diagnosed with arterial hypertension. Consequently, several 12-lead ECGs were performed, but none raised the suspicion of BrS. A positive ajmaline test confirmed a BrS diagnosis. The patient was inducible during EPS (Figure 3). Thus, the patient underwent an ICD implantation, AS ablation (Figure 3), and was referred for genetic counseling. Genetic testing revealed the variant c.4700_4701del (p.Phe1567Cysfs^*^221) in a single copy of the *SCN5A* gene (Figure 2).

**FIGURE 4:**
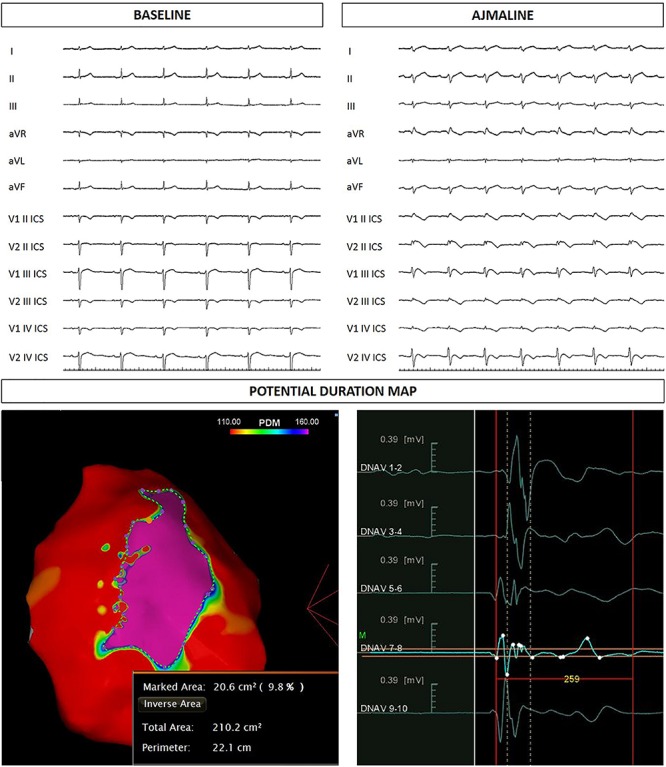
Sister of the proband: ECG at baseline and after ajmaline administration. Epicardial arrhythmogenic substrate.

The proband’s 22-year-old sister experienced syncope after gastroenteritis at the age of 20 years. She also experienced recurrent unexplained syncope episodes. Consequently, several 12-lead ECGs were performed, but none raised the suspicion of BrS. A positive ajmaline test confirmed a BrS diagnosis. For these reasons she received ICD. AS ablation was performed (Figure 4). Genetic testing revealed the variant c.4700_4701del (p.Phe1567Cysfs^*^221) in a single copy of the *SCN5A* gene.

The proband’s mother does not carry the familial variant in the *SCN5A* gene and any kind of consanguinity with her husband was excluded.

## Discussion

Many studies have described single copy *SCN5A* frameshift mutations in BrS (Probst et al., 2009; Dolz-Gaiton et al., 2013). It is known that *SCN5A* frameshift mutations can cause BrS alone or in compound heterozygosity with a missense mutation (Barajas-Martinez et al., 2013). Our hypothesis is that the novel frameshift mutation herein described is responsible for the proband’s clinical picture characterized by a complex arrhythmic phenotype.

Brugada syndrome clinical expression varies from totally asymptomatic to life-threatening arrhythmias. BrS patients harboring a compound heterozygous mutation of the *SCN5A* gene seem to be affected by the worst clinical picture (Sacilotto et al., 2017). However, a possible rescue effect due to the different contributions of two mutations in the same gene has been reported (Nunez et al., 2013).

A relationship between syncopal episodes and pediatric arrhythmias was known even before the discovery of BrS (Romano et al., 1963). Further studies have highlighted the frequency of supraventricular arrhythmias in patients affected by BrS (Liu et al., 2015). A clinical study involving 46 BrS patients (39 male and 7 female) showed that 5 (3 male and 2 female) exhibited supraventricular tachycardias (Liu et al., 2015). Another clinical study (Hasdemir, 2016) emphasized the prevalence of atrial arrhythmias in BrS patients. In fact, mutations in the *SCN5A* gene have been found responsible for atrial fibrillation, Long QT syndrome type 3, sick sinus syndrome type 2, idiopathic ventricular fibrillation, and heart block type 1A (Olson et al., 2005). While atrial fibrillation is the most common arrhythmia to overlap with BrS, cases of supraventricular tachycardia have been described (Milanesi et al., 2015). An article by Hasdemir et al. (2015) studied 96 patients with AVNRT screened for BrS. The result was that 26 out of 96 patients exhibited a BrS type 1 pattern after ajmaline challenge. Genetic testing was then performed on these patients exhibiting a dual phenotype, revealing mutations in genes known or suspected to be responsible for sodium channel loss-of-function (Hasdemir et al., 2015). In fact, several familial forms of AVNRT have been reported (Hayes et al., 2004; Namgung et al., 2012). Thus, the overlap between AVNRT and BrS may well be underappreciated.

*SCN5A* mutations have been described as causative of a variety of pathologies. *SCN5A* missense mutations can be associated with BrS or atrial fibrillation, while frameshift mutations can result in pathologies including BrS and AVNRT (Chen et al., 1998). While it is certain that *SCN5A* mutations can cause BrS, it is less clear whether the *SCN5A* mutation described herein could be responsible for the other phenotypes. Variants in genes not tested for in our patients have been associated with AVNRT (Andreasen et al., 2018). Thus, we cannot rule out the existence of AVNRT-causing variants that were not detected in this study. Additionally, we cannot exclude mitochondrial DNA variations between the proband and his sister that may account for variations in phenotype, as mitochondrial and nuclear genomes may cross-regulate each other (Kim et al., 2018). Regardless, the novel mutation herein described should be further investigated as a cause of BrS and possibly the other pathologies presented. Finally, BrS arrhythmogenic substrate ablation is a novel approach for this disease and long-term follow-up data are not yet available.

## Concluding Remarks

In this report, we documented for the first time the novel inherited frameshift mutation c.4700_4701del (p.Phe1567Cysfs^*^221) in a single copy of the *SCN5A* gene and its association with BrS. Further studies are needed to confirm this observation and to assess the clinical relevance of this mutation, in terms of risk-stratification.

## Ethics Statement

Informed consent of human subjects included in this case series report was obtained. The procedures employed were reviewed and approved by the local Ethics Committee.

## Author Contributions

EM, GC, GV, MC, VM, LG, FG, AP, MS, PP, CD, SB, MF, VS, and CP collected the data. EM and MM wrote the manuscript. All authors interpreted the results, critically reviewed the manuscript, and approved the final version.

## Conflict of Interest Statement

The authors declare that the research was conducted in the absence of any commercial or financial relationships that could be construed as a potential conflict of interest.

## Footnotes

1 https://databases.lovd.nl/shared/variants/0000371905
